# The first robotic transanal minimally invasive surgery in Ireland: a case-based review

**DOI:** 10.1007/s11845-021-02645-9

**Published:** 2021-05-12

**Authors:** Enda Hannan, Gerard Feeney, Mohammad Fahad Ullah, Kamran Amin, John Calvin Coffey, Colin Peirce

**Affiliations:** 1 Department of Colorectal Surgery, University Hospital Limerick, Limerick, Ireland; 2 School of Medicine, University of Limerick, Limerick, Ireland

**Keywords:** Rectal cancer, Robotic surgery, TAMIS, Transanal minimally invasive surgery, Transanal surgery

## Abstract

Transanal minimally invasive surgery (TAMIS) has gained worldwide acceptance as a means of local excision of early rectal cancers and benign rectal lesions. However, it is technically challenging due to the limitations of rigid laparoscopic instruments in the narrow rectal lumen. Robotic platforms offer improved ergonomics that are valuable in operative fields with limited space. Robotic TAMIS represents an exciting new development that may be more versatile than traditional TAMIS. In this review, we describe the first case of robotic TAMIS performed in our country and a review of current literature on the technique.

## Introduction

Due to morbidity associated with total mesorectal excision (TME), transanal approaches have been developed to treat early-stage rectal cancers and benign lesions [1]. However, conventional transanal excision (TAE) using anoscopic instrumentation is challenging due to inadequate exposure and visibility within the rectal lumen, compromising the ability to obtain adequate oncologic resection [2]. Transanal endoscopic microsurgery (TEMS) was first described by Buess in 1983 to overcome these challenges and uses pneumorectum, an operative microscope and angulated instruments to perform full-thickness excision of rectal lesions [1, 2]. This has been shown to significantly improve oncological outcomes and specimen quality compared with TAE, while also allowing access to more proximal lesions [3]. Despite this, adoption of TEMS in clinical practice has been limited [2, 3]. This is due to a steep learning curve, availability of costly specialised instrumentation and limited training opportunities in this technique [3]. TEMS is also not feasible for lesions close to the anal verge [3].

Transanal minimally invasive surgery (TAMIS) was introduced as an evolution of TEMS, whereby traditional laparoscopic instruments are inserted via a multi-access transanal port to perform local excision [3, 4]. This technique has gained worldwide popularity, providing the same quality of resection as TEMS without the cost and steep learning curve [3, 4]. However, TAMIS is not without limitations. Laparoscopic instruments are limited by their rigid design and inability to fully articulate. These restrictions become more pronounced when operating in small spaces such as the rectal lumen, where clashing of instruments and restrictive working angles act as barriers to performing safe dissection [4].

Robotic surgical platforms were developed to overcome the limitations of laparoscopic surgery by offering a stable 3-dimensional view, improved ergonomics and greater range of motion [4, 5]. Robotic surgery has proven to be particularly beneficial in areas with limited space, such as the pelvis and mediastinum [5]. Robotic TAMIS (R-TAMIS) was described to address the shortcomings of laparoscopic TAMIS (L-TAMIS) and has since been reported favourably in case reports and small case series [5]. However, R-TAMIS remains a novel procedure that is only performed in a small number of specialised centres [1–5]. In this report, we describe our first experience with R-TAMIS. To our knowledge, this is the first such case performed in Ireland.

## Case presentation

**Fig. 1:**
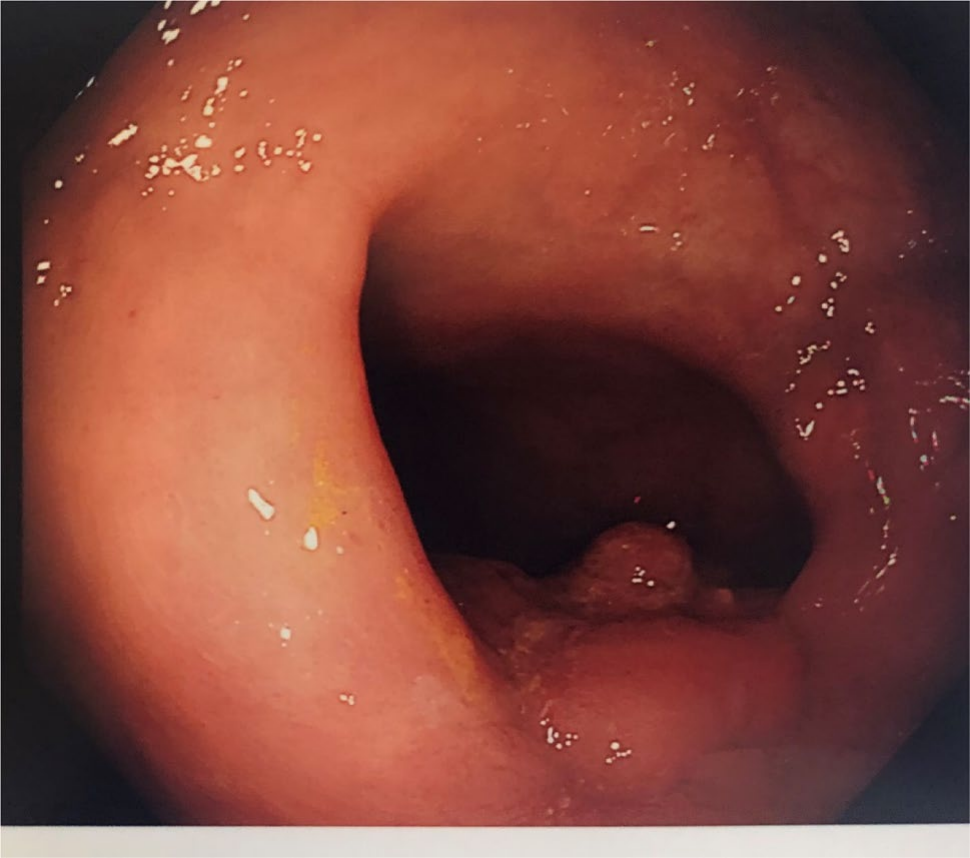
Sessile polyp located posteriorly 8 cm from the anal verge at the second rectal valve

A 45-year-old female with no significant medical history attended for an urgent colonoscopy to investigate rectal bleeding. This demonstrated a sessile polyp with a diameter of 4 cm located posteriorly at the second rectal valve approximately 8 cm from the anal verge (Fig. 1 ). A partial polypectomy was performed, and the specimen was sent for histological analysis, which reported a tubulovillous adenoma (TVA) with high grade dysplasia (HGD) and features suspicious for malignancy. Staging magnetic resonance imaging (MRI) of the pelvis noted a mid-rectal lesion suspicious for T1 or possibly T2 invasion without mesorectal lymphadenopathy. Computed tomography (CT) of the thorax, abdomen and pelvis did not detect potential metastatic disease. Following discussion at the gastrointestinal multidisciplinary meeting (MDM) and consultation with the patient the decision to proceed R-TAMIS using the da Vinci® Xi dual console robotic surgical system (Intuitive Surgical Inc, Sunnyvale, CA, USA) was made.

The patient was fully informed of the risks and benefits of this approach, including the potential necessity to proceed to an anterior resection with total mesorectal excision (TME) should the final histological result deem this necessary. The patient was also made aware that this would represent the first time this procedure had been performed in our country, and that the option of an anterior resection was also available. Following this, the patient opted to proceed with R-TAMIS. The procedure was performed by a fellowship-trained consultant colorectal surgeon on the specialist division of the medical register who had completed a proctorship programme in robotic colorectal surgery and achieved certification with the European Association of Robotic Colorectal Surgery [6]. The surgeon had also performed the required number of cases for competency in L-TAMIS [1].

After completion of mechanical bowel preparation, the procedure was commenced under general anaesthetic with the patient in the lithotomy position so that the lesion would be seen at the 6 o’clock position intraoperatively. Three robotic arms were utilised via 8-mm robotic trocars placed into the GelPOINT™ Path Transanal Access Platform (Applied Medical Inc., Rancho Santa Margarita, CA, USA), which was inserted into the anal canal and suture anchored to the surrounding skin. The robot system was docked from the left side of the patient. The first robotic arm held a fenestrated grasper with bipolar diathermy, the second the robotic camera and the third a curved scissors with monopolar diathermy. A further assistant port, an 8-mm AirSeal® trocar (CONMED, Largo, FL, USA) was used to provide suction, irrigation, traction and to aid with specimen extraction. Pneumorectum of 15 mmHg was established.

**Fig. 2:**
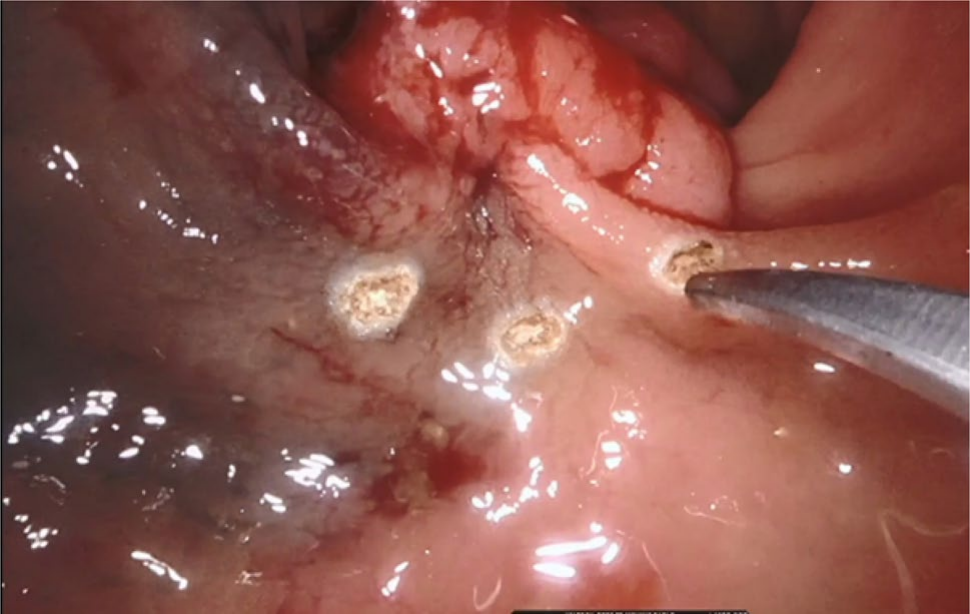
Circumferential marking of resection margins

**Fig. 3:**
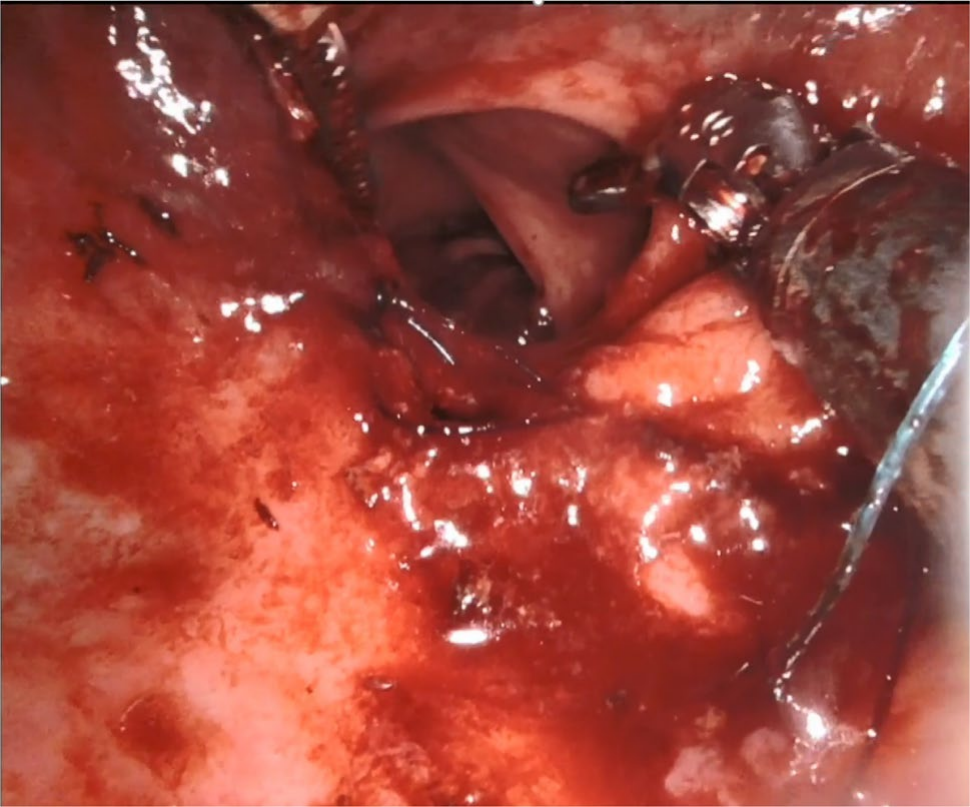
Assessing luminal patency post suturing of the defect

After visualisation of the lesion, electrocautery was used to mark adequate excision margins circumferentially (Fig. 2 ). Full-thickness excision of the lesion was performed, ensuring not to handle the lesion directly and risk fragmentation of the specimen. The presence of mesorectal fat in the base of the wound confirmed a full-thickness rectal excision. Haemostasis was carefully maintained throughout the procedure. For repair of the defect, the monopolar scissors were replaced with a robotic needle holder, and a 3.0 V-Loc™ barbed absorbable suture was inserted via the assistant port which was then mounted on the robotic needle holder. The suture was placed at the proximal apex of the wound and subsequently locked by placing the needle through the loop at the end of the thread and securing the resulting knot. The remainder of the defect was then repaired transversely in a continuous fashion. Following this, the lumen was assessed to ensure patency and that no stenosis had occurred (Fig. 3 ). The specimen was then placed in the GELPOINT path port, the robot undocked and the specimen retrieved following removal of the GELPOINT path seal and sent for histopathological analysis. Intraoperative blood loss was minimal.

The patient had an uneventful recovery and was discharged the day after surgery. The final histopathological diagnosis was a pT1 invasive moderately differentiated adenocarcinoma arising in a TVA with HGD with clear lateral margins, a deep margin of 6 mm and no poor prognostic features. At postoperative discussion at the gastrointestinal MDM, a consensus was reached that no further treatment was required given the favourable histological features of the excised lesion. The patient remains well at routine follow-up and is undergoing standard postoperative surveillance for rectal cancer.

## Discussion

This report presents the first experience with R-TAMIS in our institution, a tertiary referral university teaching hospital. To our knowledge, this represents the first time that such a technique has been performed in our country. This is of significant importance, as it offers a novel approach to early-stage rectal malignancy and benign rectal neoplasms that was previously unavailable to our patients. R-TAMIS serves as an evolution of traditional L-TAMIS, with the characteristics of the robotic platform allowing the surgeon to perform more intricate surgery with greater ease within the restrictions of the narrow rectal lumen which cannot be achieved by rigid laparoscopic instruments [1–5, 7]. The EndoWrist™ movement allows for improved intraluminal dexterity which, combined with a magnified 3D view, empowers the surgeon to perform transanal excision with improved precision [1, 2, 5]. The surgeon’s ability to perform intraluminal suturing is also improved compared to L-TAMIS, where closure of the defect can be challenging and time-consuming [3, 5]. These advantages offered by the robotic platform may allow lesions that previously would have been considered too challenging for L-TAMIS to be considered for a transanal approach, allowing the patient to avoid the considerable risk of morbidity and mortality associated with an anterior resection for a lesion that can be treated curatively by local excision [3]. Our results for this first experience with R-TAMIS are promising, and we are strongly encouraged by this initial outcome.

The literature on R-TAMIS remains relatively sparse, and this report provides valuable evidence that further serves to validate the technique. However, the evidence that currently exists is strongly encouraging [1–5, 7]. The technique was first demonstrated by Atallah in 2011 in a cadaveric model, a surgeon who is very well renowned in this field having described L-TAMIS in 2009 [1]. With recognition of the limitations of the laparoscopic approach, two tasks were assessed, the full-thickness excision of rectal tissue and subsequent closure of the defect. This was successfully completed in all attempts, and it was concluded that R-TAMIS is safe, feasible and effective. Atallah subsequently performed the first R-TAMIS in a patient successfully, which further validated the technique [2].

Following this, Lee et al*.* retrospectively compared short-term outcomes of 21 patients managed by L-TAMIS with those of 19 that underwent R-TAMIS, demonstrating a high success rate in both cohorts with comparable outcomes [3]. Interestingly, blood loss was less in the R-TAMIS group, although the cost of surgery was greater. A wider range in duration of surgery in the L-TAMIS group was attributed to variability of body habitus and tumour location, which can limit L-TAMIS compared to the more versatile R-TAMIS. The authors concluded that R-TAMIS may facilitate transanal resection not feasible by laparoscopic approach [3]. A similarly positive experience was observed in a multicentre retrospective study, where 34 patients over 2 years had rectal lesions ranging from 2 to 15 cm from the anal verge that were up to 4.5 cm in diameter successfully resected with no intraoperative complications, with all investigating surgeons reporting that R-TAMIS was less technically challenging and had a faster learning curve than L-TAMIS [4]. Hompes et al., using a surgical glove as an access platform, also reported success with R-TAMIS in a series of 16 patients, reporting a median hospital stay of 1.3 days and a low rate of morbidity, with one patient developing a pneumoperitoneum that was managed conservatively and one patient requiring catheterization for urinary retention [7]. The largest reported series of R-TAMIS to date showed the technique to be safe in 58 patients with comparable perioperative and oncological outcomes to L-TAMIS, noting an advantage of R-TAMIS is the ability to rotate the operative field allowing the surgeon to operate on all walls of the rectum rather than a single quadrant, thus allowing the surgeon to address larger lesions in multiple quadrants [5]. Warren et al., in a technical description, reported that a particular advantage of R-TAMIS is that the stability of the robotic platform creates a clearer view that allows more precise dissection, particularly at the upper part of the lesion which is usually difficult to visualize in L-TAMIS [8].

The most frequent criticisms of robotic surgery are those of increased cost and a longer operating time compared to laparoscopic approaches as a result of the expense of acquiring and utilizing robotic surgical systems and the learning curve associated with robotic docking [4, 5]. The current literature reports that the additional cost of R-TAMIS compared to L-TAMIS is approximately €1000 per procedure [6]. This is not insignificant, but may be justified by the ergonomic advantages offered by robotic systems allowing safer and more efficient removal of rectal lesions [1–5, 7, 8]. The cost should also be considered in the context of lesions that may not be feasible for L-TAMIS, where the enhanced capabilities of R-TAMIS may allow patients to avoid a costlier and potentially much more morbid anterior resection and the increased inpatient length of stay associated with this [3, 5]. With regards to operating time, the well-recognised longer time spent in theatre in robotic operations compared to laparoscopic operations has not been replicated in the literature with regards to TAMIS. In the only comparative study, no significant difference was seen between L-TAMIS and R-TAMIS [3]. This is likely as a result of the improved intraluminal dexterity compared to L-TAMIS, which makes up for time lost in the process of setting up and docking the robot [3, 9].

In our case, a T1 cancer was treated by the R-TAMIS technique. While TME remains the gold standard curative treatment for rectal cancer, it is associated with significant risk of morbidity and debilitating effects on anorectal and urogenital function [10]. Such factors have prompted the need to individualise care and to consider if organ-preserving approaches may be appropriate [10]. In the USA and Europe, full-thickness local excision is indicated for T1N0 rectal cancers with low-risk pathological features [10]. The main concern regarding local excision is the potential under-treatment of T1 cancers that are lymph node-positive. However, it has been shown that the overall rate of nodal metastases in T1 rectal cancers is as low as 6% if there are no adverse features present [10]. It has also been demonstrated that local excision of T1 lesions does not impact cancer-specific survival compared to radical resection with TME [10]. For these reasons, TAMIS is now widely accepted as an appropriate therapy for carefully selected T1 rectal cancers[10]

In conclusion, we report our first experience of a patient with early-stage rectal cancer successfully managed by R-TAMIS. The greater versatility of the robotic platform may allow lesions that would be considered unsuitable for L-TAMIS to be managed by local excision. Thus, R-TAMIS is an important evolution in transanal surgery which serves to allow patients to avoid the risks associated with more radical resection in early-stage rectal cancers and benign lesions.

## Abbreviations

TME: Total mesorectal excision
TAE: Transanal excision
TEMS: Transanal endoscopic microsurgery
TAMIS: Transanal minimally invasive surgery
L-TAMIS: Laparoscopic transanal minimally invasive surgery
R-TAMIS: Robotic transanal minimally invasive surgery
TVA: Tubulovillous adenoma
HGD: High grade dysplasia
MRI: Magnetic resonance imaging
CT: Computed tomography
MDM: Multidisciplinary meeting
